# *Bmal1* overexpression in suprachiasmatic nucleus protects from retinal neurovascular deficits in a mouse model of diabetes

**DOI:** 10.1007/s00125-026-06734-1

**Published:** 2026-04-24

**Authors:** Neha Mahajan, Qianyi Luo, Jodi Lukkes, Surabhi D. Abhyankar, Ashay D. Bhatwadekar

**Affiliations:** 1 Department of Ophthalmology, Indiana University School of Medicine, Indianapolis, IN USA; 2 Indiana University, Stark Neurosciences Research Institute, Indianapolis, IN USA; 3 Department of Biochemistry and Molecular Biology, Indiana University School of Medicine, Indianapolis, IN USA

**Keywords:** *Bmal1*, Circadian clock, Diabetic retinopathy, Gluconeogenesis, Glucose metabolism, Noradrenaline, Retinal deficits, Type 2 diabetes

## Abstract

**Aims/hypothesis:**

The suprachiasmatic nucleus regulates circadian rhythms and influences physiological and behavioural functions. Clock genes not only play a critical role in orchestrating circadian rhythms, but also regulate a variety of bodily functions. While *Bmal1*, a clock gene, is vital for maintaining optimal circadian rhythms, its therapeutic potential in type 2 diabetes remains unexplored.

**Methods:**

In this study, *db*/*db* mice, a well-established model of type 2 diabetes exhibiting arrhythmic behaviour and complications, were injected stereotaxically with AAV-*Bmal1* or a control virus into the suprachiasmatic nucleus to evaluate the protective effects of *Bmal1* overexpression on neurovascular deficits of type 2 diabetes. Given the complex neurovascular network and the eye’s unique accessibility as a transparent system, ocular complications were selected as a model to examine the neuronal functional, behavioural and vascular benefits of central overexpression of *Bmal1*.

**Results:**

*Bmal1* overexpression decreased the free-running period, which otherwise is lengthened in *db*/*db* mice. Retinal neuronal function was restored on the electroretinogram, along with optomotor behaviour and visual acuity enhancements. Retinal vascular deficits were also significantly reduced. Notably, *Bmal1* overexpression decreased fat content in genetically predisposed obese *db/db* mice compared with the untreated *db/db* group. As the suprachiasmatic nucleus is known to regulate hepatic glucose production via sympathetic mechanisms, glycaemic control and pyruvate tolerance tests were evaluated. Glucose homeostasis was improved in *Bmal1-*overexpressing mice, accompanied by a significant reduction in hepatic gluconeogenesis. Plasma noradrenaline (norepinephrine) and liver tyrosine hydroxylase levels were reduced, indicating a protective regulation of adrenergic signalling.

**Conclusions/interpretation:**

Our study highlights the therapeutic potential of central overexpression of a clock gene, *Bmal1*, to mitigate metabolic and neurovascular deficits of type 2 diabetes, offering a compelling framework for incorporating circadian rhythms into managing diabetes and its complications.

**Graphical Abstract:**

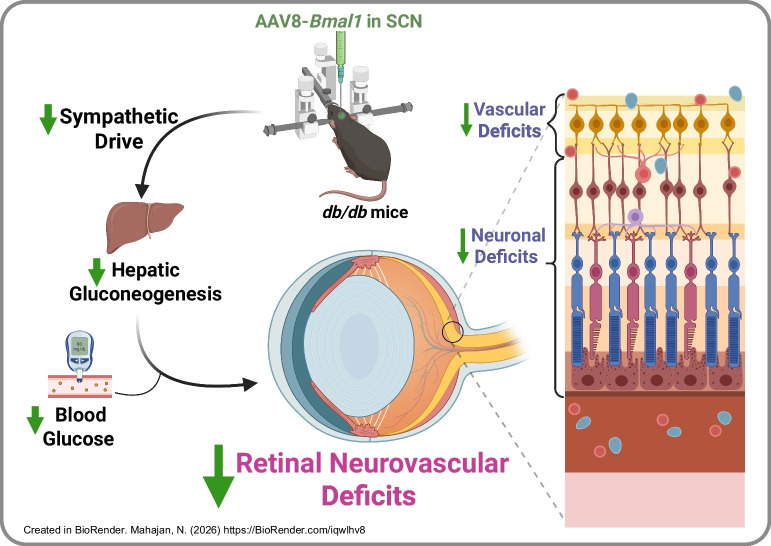

**Supplementary Information:**

The online version of this article (10.1007/s00125-026-06734-1) contains peer-reviewed but unedited supplementary material.


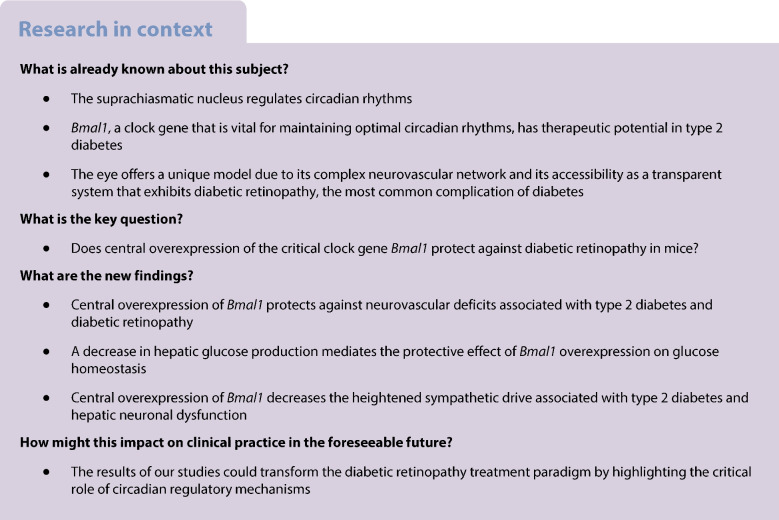


## Introduction

In mammals, the suprachiasmatic nucleus (SCN) region of the hypothalamus serves as a master pacemaker, coordinating the circadian rhythms in peripheral tissues to regulate the 24 h rhythms of physiological functions [1]. Transcriptional and translational feedback loops drive these circadian oscillations at the cellular level. The transcription factor brain and muscle ARNT-like protein 1 (BMAL1) interacts with the circadian locomotor output cycles kaput (CLOCK) to form a heterodimer, which mediates the transcription of downstream negative regulators, Period (*Per*) and Cryptochrome (*Cry*) genes. The PER/CRY heterodimer inhibits the expression and transcriptional activity of BMAL1/CLOCK [2]. Circadian rhythm misalignment, as seen in shift work and other lifestyle factors, raises the risk of conditions such as cancer, cardiovascular diseases and metabolic issues. Disturbed circadian rhythms and their molecular mechanisms also serve as pathophysiological factors in obesity, weight gain, hypertension, cardiovascular diseases and type 2 diabetes, which are key pillars of an individual’s overall cardiometabolic health [3]. Targeting circadian rhythm as a treatment paradigm through various modalities, such as clock gene modulation, light therapy, sleep interventions and feeding interventions, has shown increasing success in both preclinical and clinical studies, as reviewed previously [4].

*BMAL1* is the only non-redundant gene in the central circadian clock, increasing its significance in various circadian rhythm-focused studies [5]. Several studies have linked *Bmal1* abnormal expression patterns to metabolic complications, including type 2 diabetes. *Bmal1* deletion has been shown to promote type 2 diabetes, obesity, lipogenesis, and pancreatic beta cell impairment [6–8]. Conversely, pancreatic beta cell-specific overexpression of *Bmal1* was found to be protective against obesity-induced glucose intolerance, and mice overexpressing *Bmal1* in the same study exhibited improved glucose-stimulated insulin release [9]. While there is growing evidence highlighting the significant potential of *Bmal1* in the management of diabetes, the protective effect of central *Bmal1* overexpression on the neurovasculature in diabetes remains poorly understood. Therefore, we have employed a novel therapeutic strategy that involves overexpressing clock gene *Bmal1* centrally in the SCN via stereotaxic delivery.

The eye is uniquely suited to the study of neurovascular complications of diabetes due to the combination of a transparent window and an intricate network of retinal neurons and vasculature, making it an ideal tissue system to assess the protective effects of *Bmal1* overexpression. Additionally, the eye exhibits the most common complication of diabetes, diabetic retinopathy. It is noteworthy that the mammalian retina also possesses an autonomous circadian clock system that is independent of the SCN [10]; previously, we reported that circadian clock disruption negatively affects visual function in mice [11]. Several studies have demonstrated adverse effects of *Bmal1* deletion on retinal cone and rod cells, which could accelerate retinal microvascular and macrovascular injuries [12–14]. Therefore, in this study, we assessed the effects of central *Bmal1* overexpression on retinal neurovascular deficits in long-standing diabetes.

## Methods

### Animals

All the experiments followed the NIH Guiding Principles in the Care and Use of Animals (https://grants.nih.gov/grants/olaw/guide-for-the-care-and-use-of-laboratory-animals.pdf) and the Association for Research in Vision and Ophthalmology’s Statement for the Use of Animals in Ophthalmic and Vision Research (https://www.arvo.org/uploads/files/general/Advocacy/Animals-in-research/2024-arvo-statement-for-the-use-of-animals-in-ophthalmic-and-vision-research.pdf). The Institutional Animal Care and Use Committee at Indiana University (Indianapolis, IN, USA) approved the animal protocol (no. 23053).

Six-week-old B6.BKS(D)*-Lepr*^db^/J male mice (an animal model for type 2 diabetes; *db*/*db*) and *Lepr*^db^/^+^ *db*/*m* (heterozygotes; *db*/*m*) male mice (stock number 000697; genetic background 000664 C57BL/6J; https://www.jax.org/strain/000697) were procured from the Jackson Laboratory (Bar Harbour, ME, USA) and housed in the animal care facility at Glick Eye Institute, Indiana University. Most of the studies, including euthanasia, were performed at zeitgeber time 7 (ZT7) unless otherwise specified below. Additional details on animal husbandry as per ARRIVE guidelines [15] are provided in the electronic supplementary material (ESM) Methods.

### AAV-*Bmal1* delivery to the SCN and validation of *Bmal1* overexpression

AAV8-*Bmal1* (referred to as AAV-*Bmal1* here onwards) and a control virus were designed by and procured from the Ocular Gene Therapy Core at the University of Florida, Gainesville, FL, and the viruses were introduced stereotaxically into the SCN of 8-week-old mice. After successfully validating the SCN deliveries (ESM Fig. 1a), the mice were divided into the following groups: (1) *db*/*m* + NV (control mice/no virus); (2) *db*/*m* + Cont (*db*/*m* with adeno-associated virus [AAV] only); (3) *db*/*m* + *Bmal* (*db*/*m* with AAV-*Bmal1*); (4) *db*/*db* + NV (diabetic control mice/no virus); (5) *db*/*db* + Cont (*db*/*db* with AAV only); and (6) *db*/*db* + *Bmal* (*db*/*db* with AAV-*Bmal1*). All the mice were housed for 6 months after the injections, and then used in the experiments described below. Additionally, *Bmal1* overexpression was checked in *db*/*m* + *Bmal* and *db*/*db* + *Bmal* groups at study termination [16]. There was a significant increase (*p*=0.05) in *Bmal1* mRNA (*n*=3) in the SCN region compared with the non-SCN region in the *db*/*db* + *Bmal* group (98.19% in the SCN region, compared with 1.81% in the non-SCN region), suggesting consistent overexpression of *Bmal1* in the SCN during the course of diabetes (ESM Fig. 1b). Additional details are provided in ESM Methods.

### Echo MRI

Body composition was assessed using an EchoMRI body composition analyser (model number E26-290-RMT; EchoMRI, Houston, TX, USA) between ZT3 and ZT4. Additional details are provided in ESM Methods.

### Wheel running activity

Wheel running activity was measured as described previously [11, 17]. Additional details are provided in ESM Methods.

### Neuronal function using electroretinogram

Retinal function was measured using scotopic electroretinograms (ERGs) (LKC Technologies, Gaithersburg, MD, USA) at flash intensities of 0.025, 0.25 and 2.5 cd × s/m^2^, and the data were analysed for a- and b-wave amplitudes and implicit times. Additional details are provided in ESM Methods.

### Optomotor response behaviour

Spatial vision, i.e. optomotor response behaviour (OMR), was measured by assessing the spatial frequency threshold for optometer response using an OptoMotry device (CerebralMechanics, Lethbridge, AB, Canada) as described in ESM Methods.

### Immunohistochemistry for tyrosine hydroxylase

The liver samples were fixed in 4% paraformaldehyde, paraffin-embedded, and subsequently stained using an anti-tyrosine hydroxylase (TH) antibody (catalogue number AB152, MilliporeSigma). Additional details are provided in ESM Methods.

### Vascular deficits

Whole eyes fixed in 4% paraformaldehyde were processed for trypsin digestion, as described previously [18]. Additional details are provided in ESM Methods.

### Quantification of noradrenaline levels using ELISA

Plasma noradrenaline (norepinephrine) levels were quantified using a commercially available ELISA kit 9 (no. 3836; Novus Biologicals, Centennial, CO, USA) according to the manufacturer’s guidelines. Additional details are provided in ESM Methods.

### IPGTT

After fasting for 4 h, the IPGTT was performed as described in ESM Methods.

### IPITT

For the IPITT, the animals were fasted for 2 h, and testing was performed as described in ESM Methods.

### Intraperitoneal pyruvate tolerance test

Following an overnight fast of >16 h, an intraperitoneal pyruvate tolerance test (IPPTT) was performed as described in ESM Methods.

### Statistical analysis

All data are expressed as means ± SEM. The data were analysed using GraphPad Prism version 10.0.0 for Windows (www.graphpad.com). The Brown–Forsythe and Welch ANOVA test followed by an unpaired *t* test with Welch’s correction was used for wheel running activity, ERG, vascular deficits, Echo MRI, the AUC analysis for IPGTT, IPITT and IPPTT, and the noradrenaline ELISA. One-way ANOVA followed by Tukey’s post hoc test was used to analyse OMR data, and either one-way or two-way ANOVA was used for IPGTT, IPITT and IPPTT analysis, followed by Tukey’s post hoc test or the Brown–Forsythe and Welch ANOVA test. Data were considered statistically significant when the *p* value was less than 0.05.

## Results

### *Bmal1* overexpression shortened the duration of free-running periods in *db*/*db* mice

**Fig. 1:**
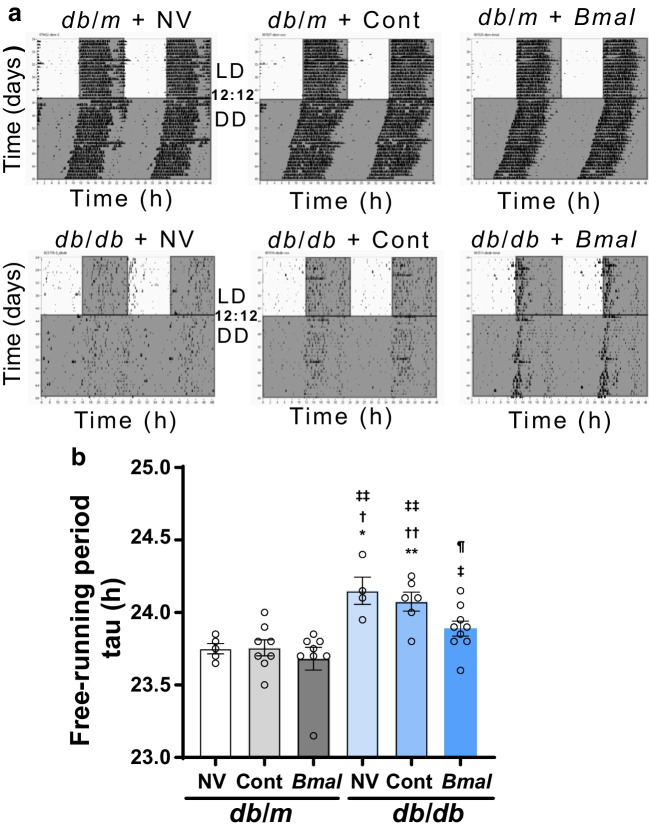
*Bmal1* overexpression in the SCN shortens the duration of free-running periods. (**a**) Representative double-plotted actograms of wheel running activity under 12 h light/12 h dark conditions (LD12:12) and constant dark conditions (DD). (**b**) Free-running period (symbolised as tau). The numbers of animals in each group were as follows: *db*/*m* + NV: *n*=5; *db*/*m* + Cont: *n*=8; *db*/*m* + *Bmal*: *n*=8; *db*/*db* + NV: *n*=4; *db*/*db* + Cont: *n*=6; *db*/*db* +* Bmal*: *n*=9. The data are means ± SEM and were analysed using Brown–Forsythe and Welch ANOVA tests followed by unpaired *t* test with Welch’s correction. Statistically significant differences are indicated by different symbols according to comparator group: vs *db*/*m* + NV: *p<0.05, **p<0.01; vs *db*/*m* + Cont: ^†^*p*<0.05, ^††^*p*<0.01; vs *db*/*m* + *Bmal*: ^‡^*p*<0.05, ^‡‡^*p*<0.01; vs *db*/*db* + Cont, ^¶^*p*<0.05

The animals were assessed for voluntary wheel running activity and free-running period after 8 weeks of *Bmal1* overexpression (Fig. 1a). The numbers of mice in each group were as follows: *db*/*m* + NV: *n*=5; *db*/*m* + Cont: *n*=8; *db*/*m* + *Bmal*: *n*=8; *db*/*db* + NV: *n*=4; *db*/*db* + Cont: *n*=6; *db*/*db* + *Bmal*: *n*=9. We observed that *db*/*db* + NV mice and *db*/*db* + Cont groups have reduced total activity compared with *db*/*m* + NV mice (26.5-fold decrease, *p*=0.0032, for *db*/*db* + NV mice; 36.4-fold decrease, *p*=0.0033, for *db*/*db* + Cont mice; *W*_(5,15.66)_ = 49.49, *p*<0.001), and there was no effect of *Bmal1* overexpression (ESM Fig. 2). On the other hand, the free-running periods measured in the absence of external light cue or constant dark phase were significantly lengthened in *db*/*db +* NV mice (24.15±0.09) and *db*/*db* + Cont mice (24.08±0.06) compared with *db*/*m* + NV mice (23.75±0.03) (*p*=0.0173 and *p*=0.0028, respectively; *F*_(5,25)_ = 7, *p*=0.002) (Fig. 1b). Interestingly, *Bmal1* overexpression in *db*/*db* mice led to a significantly shorter free-running period when compared with *db*/*db* + Cont mice (*p*=0.0493) (Fig. 1b), suggesting an overall improvement in endogenous period.

### *Bmal1* overexpression improved OMR in *db*/*db* mice

**Fig. 2:**
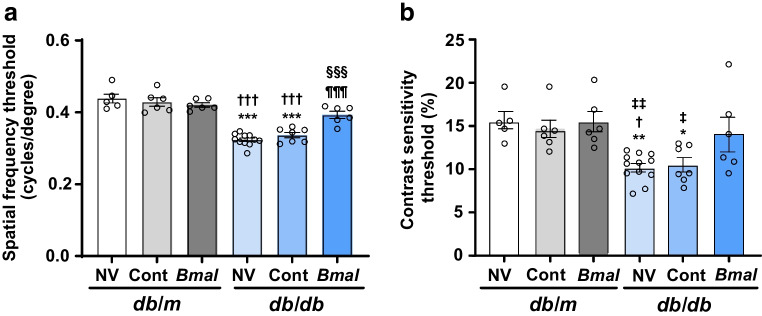
*Bmal1* overexpression in the SCN improved visual acuity behaviour. (**a**) *db*/*db* mice overexpressing *Bmal1* showed a significant increase in the spatial frequency threshold measured using an OMR tracking system. (**b**) Contrast sensitivity remained unchanged when *Bmal1* was overexpressed. The numbers of animals in each group were as follows: *db*/*m* + NV: *n*=5; *db*/*m* + Cont: *n*=6; *db*/*m* +* Bmal*: *n*=6; *db*/*db* + NV: *n*=11 for (**a**) and *n*=12 for (**b**); *db*/*db* + Cont: *n*=7; *db*/*db* +* Bmal*: *n*=6. The data are means ± SEM and were analysed using one-way ANOVA followed by Tukey’s post hoc test. Statistically significant differences are indicated by different symbols according to comparator group: vs *db*/*m* + NV: *p<0.05, **p<0.01, ***p<0.001; vs *db*/*m* + Cont: ^†^*p*<0.05, ^†††^*p*<0.001; vs *db*/*m* + *Bmal*: ^‡^*p*<0.05, ^‡‡^*p*<0.01; vs *db*/*db* + NV: ^§§§^*p*<0.001; vs *db*/*db* + Cont: ^¶¶¶^*p*<0.001

Next, we analysed the beneficial effects of SCN *Bmal1* overexpression on optomotor behaviour. We studied OMR tracking to check whether *Bmal1* influences visual performance. These studies were conducted at ZT7. The numbers of mice in each group were as follows: *db*/*m* + NV: *n*=5; *db*/*m* + Cont: *n*=6; *db*/*m* +* Bmal*: *n*=6; *db*/*db* + NV: *n*=11 for spatial frequency, *n*=12 for contrast sensitivity; *db*/*db* + Cont: *n*=7; *db*/*db* +* Bmal*: *n*=6. The spatial frequency threshold was significantly reduced in the *db*/*db* mice (1.35-fold, *p*<0.001) and *db*/*db* + Cont mice (1.3-fold, *p*<0.001) compared with *db*/*m* mice, and was significantly increased in the *db*/*db* + *Bmal* mice compared with the *db*/*db* + NV mice and *db*/*db* + Cont mice (1.2-fold, *p*<0.001 and 1.16-fold, *p*=0.0008, respectively; *F*_(5,35)_ = 36.63, *p*<0.001) (Fig. 2a). The contrast sensitivity was significantly lower in the *db*/*db* + NV mice (10.25±0.4674, *p*=0.0077, and *db*/*db* + Cont mice (10.53±0.8058, *p*=0.0293) compared with the *db*/*m* + NV mice (15.66±1.089). *Bmal1* overexpression slightly improved the contrast sensitivity (14.08±1.916); however, the difference was non-significant (*F*_(5,36)_ = 6.12, *p*=0.0003) (Fig. 2b).

### *Bmal1* overexpression improved retinal function in *db*/*db* mice

**Fig. 3:**
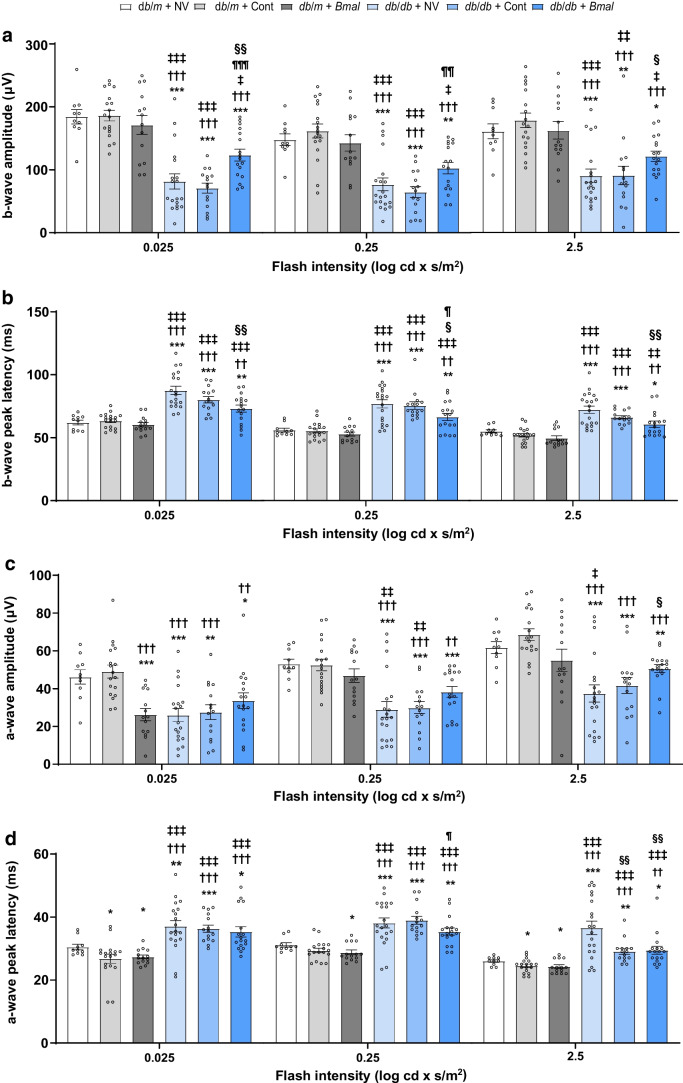
*Bmal1* overexpression in the SCN improved neuronal function. (**a**) Scotopic b-wave amplitude quantification, (**b**) b-wave peak latency time, (**c**) scotopic a-wave amplitude quantification, and (**d**) a-wave peak latency time. The numbers of animals in each group were as follows: *db*/*m* + NV: *n*=10; *db*/*m* + Cont: *n*=18; *db*/*m* +* Bmal*: *n*=14; *db*/*db* + NV: *n*=20 (except for the sub-part of (**a**) relating to the amplitude 0.025 log cd × s/m^2^, for which *n*=19); *db*/*db* + Cont: *n*=15; *db*/*db* +* Bmal*: *n*=17. The data are means ± SEM and were analysed using Brown–Forsythe and Welch ANOVA tests followed by unpaired *t* test with Welch’s correction. Statistically significant differences are indicated by different symbols according to comparator group; vs *db*/*m* + NV: *p<0.05, **p<0.01, ***p<0.001; vs *db*/*m* + Cont: ^††^*p*<0.01, ^†††^*p*<0.001; vs *db*/*m* + *Bmal*: ^‡^*p*<0.05, ^‡‡^*p*<0.01, ^‡‡‡^*p*<0.001; vs *db*/*db* + NV: ^§^*p*<0.05, ^§§^*p*<0.01; vs *db*/*db* + Cont: ^¶^*p*<0.05, ^¶¶^*p*<0.01, ^¶¶¶^*p*<0.001

Further, we examined the effect of *Bmal1* overexpression on retinal function using an ERG. The ERG results originate from currents within the eye caused by light-induced activity in neurons, glia and retinal pigment epithelial cells. These can be broken down into wave components associated with specific cells or groups of cells. Photoreceptors generate a negative a-wave, while a positive peak from the b-wave indicates less intense stimuli than the a-wave and reflects bipolar cell activity due to the convergence of rods onto bipolar cells [19]. Mice were dark-adapted for ERG response under scotopic conditions, and the studies were conducted at ZT7. The numbers of mice in each group were as follows: *db*/*m* + NV: *n*=10; *db*/*m* + Cont: *n*=18; *db*/*m* + *Bmal*: *n*=14; *db*/*db* + NV: *n*=20, except for scotopic b-wave 0.025 log cd × s/m^2^ amplitude where *n*=19; *db*/*db* + Cont: *n*=15; *db*/*db* + *Bmal*: *n*=17. There was a significant reduction in the b-wave amplitude at all three intensities in the *db*/*db* + NV mice (2.25-fold decrease at 0.025 log cd × s/m^2^, *p*<0.001; 1.92-fold decrease at 0.25 log cd × s/m^2^, *p*<0.001; 1.78-fold decrease at 2.5 log cd × s/m^2^, *p*=0.0002) and in the *db*/*db* + Cont mice (2.6-fold decrease at 0.025 log cd × s/m^2^, *p*<0.001; 2.28-fold decrease at 0.25 log cd × s/m^2^, *p*<0.001; 1.76-fold decrease at 2.5 log cd × s/m^2^, *p*=0.0011), which was enhanced in *db*/*db* + *Bmal* mice (1.51-fold higher, *p*=0.0098, at 0.025 log cd × s/m^2^; 1.33-fold higher, *p*=0.0659, at 0.25 log cd × s/m^2^; 1.34-fold higher, *p*=0.0251, at 2.5 log cd × s/m^2^), suggesting a protective effect of *Bmal1* overexpression on inner retinal function in *db*/*db* mice (Fig. 3a). Similarly, there was also a reduction in the b-wave peak latency time in *db*/*db* + *Bmal* mice (1.19-fold decrease, *p*=0.0022, at 0.025 log cd × s/m^2^; 1.15-fold decrease, *p*=0.0226, at 0.25 log cd × s/m^2^, 1.18-fold decrease, *p*=0.0050, at 2.5 log cd × s/m^2^), which suggests a decrease in the time taken to generate the positive b-wave from the onset of stimulus (Fig. 3b). The a-wave amplitude and peak latency time, reflecting rod cell activity under scotopic conditions (and hence outer retinal function), were also improved in *db*/*db* + *Bmal* mice, but the difference was only significant at the 2.5 log cd × s/m^2^ flash intensity (1.35-fold increase, *p*=0.0142; *F*_(5.0,63.29)_ = 9.45, *p*<0.001 for a-wave amplitude; 1.24-fold decrease, *p*=0.0069; *F*_(5.0,41.57)_ = 16.90, *p*<0.001, for a-wave peak latency) (Fig. 3c,d). The ERG analysis suggested that *Bmal1* overexpression improves retinal function by enhancing bipolar cell and rod photoreceptor function.

### *Bmal1* overexpression decreased the vascular deficits in *db*/*db* mice

**Fig. 4:**
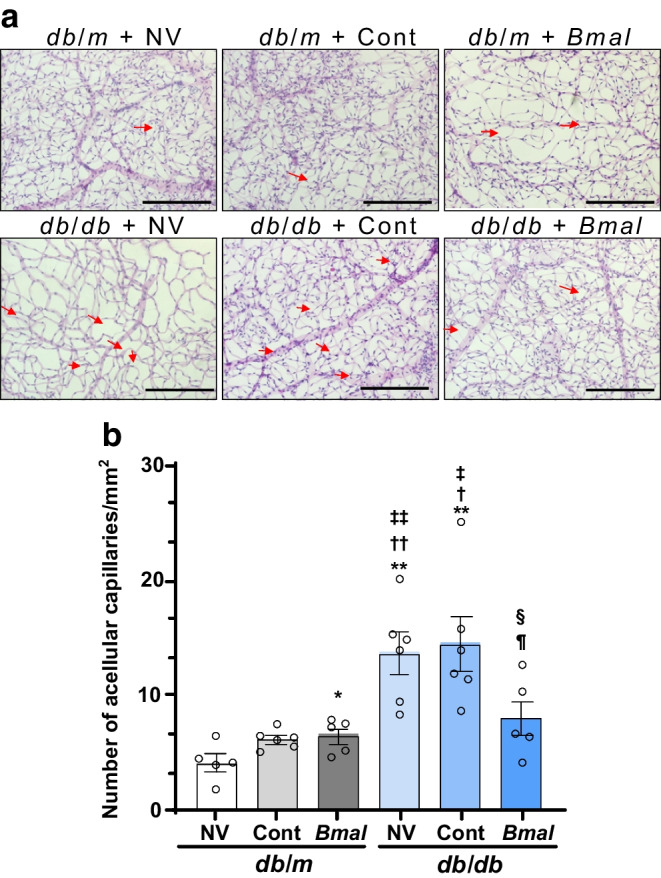
Decrease in vascular deficits in *db*/*db* mice overexpressing *Bmal1* in the SCN. (**a**) Representative images of trypsin-digested retinas. Red arrows show the changes in acellular capillary numbers. (**b**) Quantification of acellular capillary numbers. Scale bars=100 µm. The numbers of animals in each group were as follows: *db*/*m* + NV: *n*=5; *db*/*m* + Cont: *n*=6; *db*/*m* + *Bmal*: *n*=5; *db*/*db* + NV: *n*=6; *db*/*db* + Cont: *n*=6; *db*/*db* + *Bmal*: *n*=5. The data are means ± SEM and were analysed using Brown–Forsythe and Welch ANOVA tests followed by unpaired *t* test with Welch’s correction. Statistically significant differences are indicated by different symbols according to comparator group: vs *db*/*m* + NV: *p<0.05, **p<0.01; vs *db*/*m* + Cont: ^†^*p*<0.05, ^††^*p*<0.01; vs *db*/*m* + *Bmal*: ^‡^*p*<0.05, ^‡‡^*p*<0.01; vs *db*/*db* + NV: ^§^*p*<0.05; vs *db*/*db* + Cont: ^¶^*p*<0.05

After examining behavioural functional vision using OMR and retinal functions using ERG, we next examined the vascular phenotype (Fig. 4), i.e. the change in acellular capillary numbers. These studies were performed on mice after euthanasia at ZT7. The numbers of mice in each group were as follows: *db*/*m* + NV: *n*=5; *db*/*m* + Cont: *n*=6; *db*/*m* + *Bmal*: *n*=5; *db*/*db* + NV: *n*=6; *db*/*db* + Cont: *n*=6; *db*/*db* + *Bmal*: *n*=5. The *db*/*db* + NV mice and *db*/*db* + Cont mice showed a significant increase in the acellular capillaries (13.68±1.749, *p*=0.0018, and 14.47±2.339, *p*=0.0059, respectively) compared with the *db*/*m* mice (4.210±0.7398; *F*_(5.0,14.75)_ = 9.0, *p*=0.0004) (Fig. 4a,b). There was a significant reduction in the number of acellular capillaries in *db*/*db* + *Bmal* mice (8.060±1.519) compared with both *db*/*db* + NV mice and *db*/*db* + Cont mice (*p*=0.0382 and *p*=0.0496, respectively; Fig. 4b), suggesting a preventive effect on diabetic retinopathy.

### *Bmal1* overexpression improved glucose homeostasis and physiological parameters in *db*/*db* mice

**Fig. 5:**
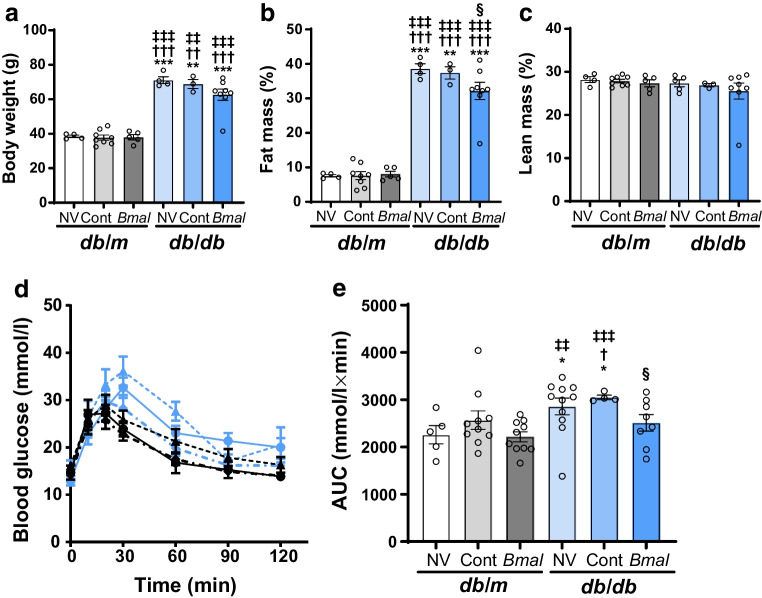
Effect of *Bmal1* overexpression in the SCN on anthropometry and glucose homeostasis. (**a**) Body weight, (**b**) fat mass and (**c**) lean mass measured using Echo MRI. (**d**) Results of the IPGTT and (**e**) corresponding AUC. The numbers of animals in each group were as follows: for (**a**–**c**): *db*/*m* + NV: *n*=4; *db*/*m* + Cont: *n*=8; *db*/*m* +* Bmal*: *n*=5; *db*/*db* + NV: *n*=4; *db*/*db* + Cont: *n*=3; *db*/*db* + *Bmal*: *n*=8; for (**d**) and (**e**): *db*/*m* + NV: *n*=5; *db*/*m* + Cont: *n*=10; *db*/*m* + *Bmal*: *n*=10; *db*/*db* + NV: *n*=11; *db*/*db* + Cont: *n*=4; *db*/*db* +* Bmal*: *n*=8. The data are means ± SEM and were analysed using Brown–Forsythe and Welch ANOVA tests followed by unpaired *t* test with Welch’s correction. Statistically significant differences are indicated by different symbols according to comparator group: vs *db*/*m* + NV: *p<0.05, **p<0.01, ***p<0.001; vs *db*/*m* + Cont: ^†^*p*<0.05, ^††^*p*<0.01, ^†††^*p*<0.001; vs *db*/*m* + *Bmal*: ^‡‡^*p*<0.01, ^‡‡‡^*p*<0.001; vs *db*/*db* + NV: ^§^*p*<0.05

While circadian rhythms, neurovascular deficits and behavioural responses were improved using our therapeutic strategy, the systemic effects of *Bmal1* overexpression were not assessed. The literature suggests that *Bmal1* knockout animals exhibit weight gain and glucose intolerance [8, 20]. To investigate the peripheral benefits of central *Bmal1* overexpression, we first evaluated the body composition of mice using Echo MRI (Fig. 5a–c). The numbers of mice in each group were as follows: *db*/*m* + NV: *n*=4; *db*/*m* + Cont: *n*=8; *db*/*m* + *Bmal*: *n*=5; *db*/*db* + NV: *n*=4; *db*/*db* + Cont: *n*=3; *db*/*db* + *Bmal*: *n*=8. The body weight was significantly higher in *db*/*db* + NV mice and *db*/*db* + Cont mice (1.84-fold, *p*=0.0002 and 1.78-fold, *p*=0.0047, respectively; *F*_(5,16.74)_ = 53.56, *p*<0.001), while *db*/*db* + *Bmal* mice had a reduced body weight compared with *db*/*db* + NV mice (1.13-fold; Fig. 5a); however, this difference was not statistically significant (*p*=0.0532). Strikingly, the fat mass was reduced significantly in *db*/*db* + *Bmal* mice compared with *db*/*db* + NV mice (1.2-fold less, *p*=0.0489; *F*_(5,15.60)_ = 93.72, *p*<0.001) (Fig. 5b). There was no observable difference in lean mass percentage among all the groups (*F*_(5,12.75)_ = 0.999, *p*=0.4563) (Fig. 5c).

Next the effect of *Bmal1* overexpression on glucose homeostasis was evaluated using an IPGTT. The numbers of mice in each group were as follows: *db*/*m* + NV: *n*=5; *db*/*m* + Cont: *n*=10; *db*/*m* + *Bmal*: *n*=10; *db*/*db* + NV: *n*=11; *db*/*db* + Cont: *n*=4; *db*/*db* + *Bmal*: *n*=8. The IPGTT curve suggested an impaired glucose clearance in *db*/*db* + NV and *db/db* + Cont mice compared with the respective *db*/*m* + NV mice (time: *F*_(6,252)_ = 79.07; *p*<0.001; treatment: *F*_(5,42)_ = 1.98, *p*=0.10; interaction: *F*_(30,252)_ = 2.26; *p*<0.001) (Fig. 5d). Further, we found higher AUCs for blood glucose in *db*/*db* + NV mice (2859±176.10 mmol/l × min) and *db*/*db* + Cont mice (3050±45.59 mmol/l × min) compared with *db*/*m* + NV mice (2260±196.60 mmol/l × min). However, *db*/*db* + *Bmal* mice showed a significant improvement in glucose homeostasis based on AUC analysis compared with *db*/*db* + Cont mice (2511±175.90 mmol/l × min, *p*=0.0182; *F*_(5,37)_ = 3, *p*=0.0258) (Fig. 5e).

We also examined insulin sensitivity using an IPITT: the *db*/*db* + *Bmal* mice showed an overall faster clearance of glucose compared with untreated *db*/*db* mice or *db*/*db* + Cont mice (time:* F*_(4,220)_=56.22; *p*<0.001; treatment: *F*_(5,55)_=6.06; *p*<0.001; interaction: *F*_(20,220)_=3.93; *p*<0.001). Determination of the AUC showed a reduction in blood glucose clearance in *db*/*db* + NV mice and *db*/*db* + Cont mice (986.7±60.73 mmol/l × min, *p*<0.001, and 1010±139.7 mmol/l × min, *p*=0.0095, respectively), and *Bmal1* overexpression did not improve insulin-dependent glucose homeostasis in *db*/*db* + *Bmal* mice (864.8±99.96 mmol/l × min; *F*_(5,24.49)_ = 7.81, *p*=0.0002) (ESM Fig. 3).

### *Bmal1* overexpression improved hepatic gluconeogenesis through noradrenaline signalling

**Fig. 6:**
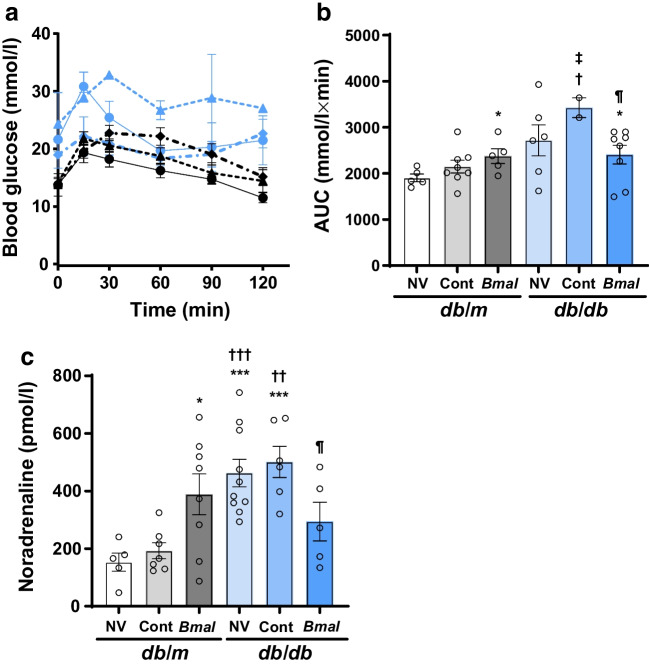
Effect of *Bmal1* overexpression in the SCN on hepatic gluconeogenesis and the sympathetic nervous system. (**a**) Results of the IPPTT and (**b**) corresponding AUC. (**c**) Plasma noradrenaline levels were quantified using ELISA. The numbers of animals in each group were as follows: for IPPTT: *db*/*m* + NV: *n*=5; *db*/*m* + Cont: *n*=8; *db*/*m* + *Bmal*: *n*=5; *db*/*db* + NV: *n*=6; *db*/*db* + Cont: *n*=2; *db*/*db* + *Bmal*: *n*=8; for noradrenaline measurement: *db*/*m* + NV: *n*=5; *db*/*m* + Cont: *n*=7; *db*/*m* +* Bmal*: *n*=8; *db*/*db* + NV: *n*=10; *db*/*db* + Cont: *n*=6; *db*/*db + Bmal*: *n*=5. The data are means ± SEM and were analysed using Brown–Forsythe and Welch ANOVA tests followed by unpaired *t* test with Welch’s correction. Statistically significant differences are indicated by different symbols according to comparator group: vs *db*/*m* + NV: *p<0.05, ***p<0.001; vs *db*/*m* + Cont: ^†^*p*<0.05, ^††^*p*<0.01, ^†††^*p*<0.001; vs *db*/*m* + *Bmal*: ^‡^*p*<0.05; vs *db*/*db* + Cont: ^¶^*p*<0.05

*Bmal1* is a well-known regulator of the central circadian clock. Our IPGTT results indicated an improvement in glucose intolerance. However, the insulin-dependent glucose homeostasis remained unchanged. *Bmal1* also regulates hepatic glucose metabolism [21]. To determine whether similar mechanisms underlie the beneficial effects of central *Bmal1* overexpression in our study, we performed an IPPTT, which is a routinely used test of hepatic gluconeogenesis. The numbers of mice in each group were as follows: *db*/*m* + NV: *n*=5; *db*/*m* + Cont: *n*=8; *db*/*m* + *Bmal*: *n*=5; *db*/*db* + NV: *n*=6; *db*/*db* + Cont: *n*=2; *db*/*db* + *Bmal*: *n*=8. The IPPTT curve showed an overall reduction in blood glucose in the *db*/*db* + *Bmal* mice (time: *F*_(5,140)_=9.98; *p*<0.001; treatment: *F*_(5,28)_=4.14; *p*=0.0061; interaction: *F*_(25,140)_=1.65; *p*=0.0365) (Fig. 6a). The estimation of AUC exhibited an increase in hepatic glucose production/gluconeogenesis in *db*/*db* + NV mice (2717±335.9 mmol/l × min, *p*=0.0597) and *db*/*db* + Cont mice (3427±214.5 mmol/l × min, *p*=0.0576) compared with *db*/*m* + NV mice (1903±82.42 mmol/l × min); this was reduced significantly in *db*/*db* + *Bmal* mice (2407±202.5 mmol/l × min, *p*=0.0367 vs *db*/*db* + Cont mice; *F*_(5,15.78)_ = 3.87, *p*=0.0176) (Fig. 6b), suggesting a direct impact of *Bmal1* overexpression in the SCN on hepatic gluconeogenesis and glucose metabolism.

Interestingly, hyperglycaemia and impaired glucose homeostasis can increase the sympathetic drive, leading to elevated adrenaline (epinephrine) levels [22]. Reciprocally, the literature also suggests that suboptimal management of diabetes could lead to an increase in plasma noradrenaline levels [23, 24]. We were intrigued by the possibility that central overexpression of *Bmal1* influences the sympathetic drive, which in turn contributes to poor blood glucose homeostasis. Therefore, plasma noradrenaline levels were quantified to understand the mechanism behind the peripheral protective effects of *Bmal1* on glucose homeostasis. The numbers of mice in each group were as follows: *db*/*m* + NV: *n*=5; *db*/*m* + Cont: *n*=7; *db*/*m* + *Bmal*: *n*=8; *db*/*db* + NV: *n*=10; *db*/*db* + Cont: *n*=6; *db*/*db* + *Bmal*: *n*=5. The noradrenaline levels were significantly higher in *db*/*db* + NV mice (462.5±47.91 pmol/l, *p*=0.001) and *db*/*db* + Cont mice (501±54.09 pmol/l, *p*=0.0006) compared with *db*/*m* + NV mice (153±31.67 pmol/l), and *Bmal1* overexpression in *db*/*db* mice reduced the circulating noradrenaline levels compared with *db*/*db* + Cont mice (294±66.95, *p*=0.0423; *F*_(5,26.12)_ = 7.06, *p*=0.0003) (Fig. 6c).

**Fig. 7:**
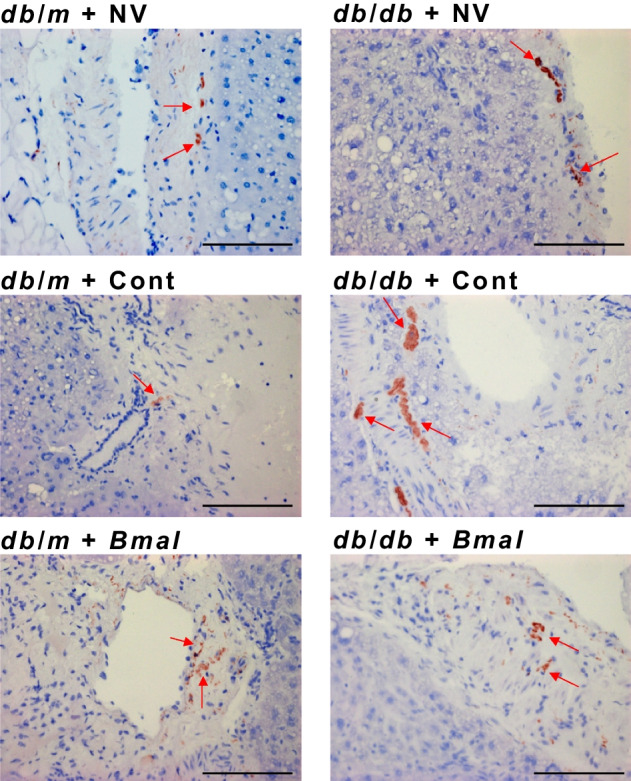
*Bmal1* overexpression in the SCN downregulates TH expression. Representative images of TH antibody-stained liver sections; red arrows indicate positive staining. Magnification: 20×; Scale bars=100 µm; *n*=5 for all the groups

TH is a rate-limiting enzyme in the synthesis of noradrenaline, thereby affecting the sympathetic drive [25, 26]. The numbers of mice in each group were as follows: *db*/*m* + NV: *n*=5; *db*/*m* + Cont: *n*=5; *db*/*m* + *Bmal*: *n*=5; *db*/*db* + NV: *n*=5; *db*/*db* + Cont: *n*=5; *db*/*db* + *Bmal*: *n*=5. We observed upregulation of TH in liver sections from the *db*/*db* + NV and *db*/*db* + Cont groups compared with *db*/*m* + NV groups (Fig. 7). This TH activation was downregulated in *db*/*db* mice overexpressing *Bmal1.*

## Discussion

This study highlights that targeting circadian rhythm-related mechanisms via *Bmal1* overexpression in the SCN protects from neurovascular deficits of the retina in type 2 diabetes. Furthermore, our findings illustrate how *Bmal1* overexpression in the SCN influences systemic physiological aspects such as sympathetic activity and glucose intolerance, a relationship that has not been investigated previously. Overall, our studies emphasise the potential of targeting the circadian rhythm for treatment of diabetes and its complications.

In mammals, circadian rhythms are regulated by a master clock in the SCN of the hypothalamus, which in turn governs the circadian clock of peripheral organs. Interestingly, recent research has demonstrated downregulation of hippocampal *Bmal1* in a streptozocin/high-fat diet-induced mouse model of diabetes [27]. In our study, the *Bmal1* overexpression in *db*/*db* mice shortened the duration of the free-running period, which otherwise increased in *db*/*db* animals. This observation is consistent with previous studies showing that *Bmal1* deletion in arginine vasopressin neurons in the dorsal SCN region prolonged the duration of the free-running period in these mice [28]. It is noteworthy that the lengthening of the period in *db*/*db* mice is due to severe diabetes, as diet-induced mouse models of diabetes also show a similar lengthening of the period; however, *ob*/*ob* mice do not exhibit period lengthening [29]. Therefore, we speculate that the beneficial effects of *Bmal1* overexpression on the duration of the free-running period in *db/db* mice may be related to improved diabetes conditions rather than the *db*/*db* phenotype.

Research has shown that mouse models of diabetes (both type 1 and type 2) show a reduction in the expression of clock genes [30–32], which contributes to the circadian arrhythmicity observed in *db*/*db* mice and leads to disrupted glycaemic control [11, 29, 33]. In our study, *Bmal1* overexpression in the SCN halted the increase in acellular capillaries, which is a hallmark of diabetic retinopathy. Also, the reduction in b-wave amplitude in *db*/*db* mice was improved after *Bmal1* overexpression in the SCN of *db*/*db* mice. Conditional *Bmal1* knockout mice also exhibit retinal deficits, specifically affecting the circadian rhythmicity of the ERG b-wave amplitude [10]. However, in our study, we do not expect *Bmal1* overexpression in the SCN to have directly affected retinal *Bmal1* or influenced ocular parameters, although we cannot exclude the potential for improved retrograde signalling.

One notable finding of our study is a decrease in hepatic glucose production, which serves as the mode of action for peripheral glucose homeostasis and has not been previously studied in this context. The liver is a principal organ for glucose storage, and disruption of liver functions has detrimental metabolic consequences. La Fleur et al showed that injecting the trans-neuronal pseudorabies virus into the liver resulted in retrograde labelling of neurons in the central nervous system [34]. Notably, the localisation of third-order neurons in the SCN illustrates the presence of anatomical pathways that enable the biological clock to influence autonomic input to the liver, emphasising the direct effects of restoring the SCN clock on liver function [34]. Moreover, sympathetic activation stimulates hepatic glucose production via gluconeogenesis and glycogenolysis, contrary to the effects of parasympathetic activation, leading to a reduction in glucose production [35, 36]. Intriguingly, we observed increased plasma levels of noradrenaline (a sympathetic neurotransmitter) in *db*/*db* mice. In contrast, *Bmal1*-overexpressing *db*/*db* mice showed a significant reduction in noradrenaline levels, suggesting impaired gluconeogenesis and glucose homeostasis in *db*/*db* mice. Published literature has also shown that noradrenaline can influence rodent hepatic circadian rhythms and clock gene expression [37].

Moreover, as we delved deeper into the sympathetic system and hepatic gluconeogenesis as the principal mechanisms underlying the SCN’s effects, we also analysed hepatic TH expression. TH is a rate-limiting enzyme for synthesising catecholamines (adrenaline, noradrenaline and dopamine) that is known to be expressed in the nerve fibres around the portal vein, bile duct or hepatic arteries of the liver. Consistent with a previous report on the higher expression of TH in the liver of obese animals [38], we also observed a higher expression of TH in *db*/*db* mice. However, overexpression of *Bmal1* in *db*/*db* mice significantly decreased TH expression. We speculate that higher hepatic TH expression, accompanied by a simultaneous increase in plasma noradrenaline levels, impairs glucose metabolism in this mouse model of diabetes.

Although the central overexpression of *Bmal1* enhanced glycaemic control, it did not impact insulin-dependent glucose clearance. This lack of change may be due to the genetic background of *db*/*db* mice (which are deficient in leptin receptors), as *Bmal1* may interact with leptin and its receptors to influence various metabolic pathways, including insulin sensitivity and weight gain [8]. It is also important to emphasise that, in the present study, overexpressing *Bmal1* in the SCN reduced fat mass in genetically predisposed *db/db* mice. Given recent advances in diabetes treatments (such as glucagon-like peptide-1 agonists and sodium–glucose co-transporter 2 inhibitors) that target central mechanisms, our approach paves the way for future pharmaceutical developments. Furthermore, while the SCN is known to influence leptin directly [39], use of leptin receptor-resistant mice allowed us to exclude leptin-mediated mechanisms, concentrating primarily on the SCN-mediated sympathetic pathway.

Most of our studies were performed at ZT7, except for body composition measurements. The rationale for performing eye measurements at ZT7 is based on our prior studies and lab data. The ERG response in *db*/*m* age-matched wild-type mice peaked between ZT3 and ZT9, with a peak closer to ZT7 [40]. Interestingly, *db*/*db* mice lack circadian rhythmicity in ERG even when they are young [40] (the circadian rhythm of activity is maintained [29]); however, as diabetes progresses over 6 months (the same timeframe as this study) in *db*/*db* mice, or due to natural ageing in the case of *db*/*m* animals, the circadian rhythmicity of ERG is lost [40]. Therefore, we do not expect a rhythmic pattern in ERG studies due to long-standing diabetes. Additionally, unpublished optokinetic studies from our lab on wild-type mice (C57BL/6J background) did not show any rhythmic changes under diurnal conditions (Mathew and Bhatwadekar). It would be interesting to conduct glucose tolerance studies and perform measurements of noradrenaline levels at different time points to understand the circadian rhythmicity of these parameters. Also, we did not quantify the food intake, which is a well-known zeitgeber for circadian rhythms. However, for now, our studies primarily focused on hepatic gluconeogenesis and the noradrenaline pathway as a peripheral mechanism to counteract the long-term effect of diabetes on retinal impairments, with the aim of establishing central *Bmal1* overexpression as a therapeutic strategy. We acknowledge the above limitations of our study, but it nonetheless provides insights into areas for future exploration.

In conclusion, our study demonstrates that therapies aimed at correcting circadian misalignment may prove beneficial in treating diabetes and its associated complications. Moreover, we found that targeting the central clock protects sympathetic nervous system activation and glycaemic control. Collectively, these findings provide a novel direction for using circadian clock genes as therapeutic targets for diabetes and its related complications.

## Supplementary Information

Below is the link to the electronic supplementary material.

ESM (PDF 1244 KB)

## Data Availability

All data supporting the findings of this study are included in the paper and the electronic supplementary information.

## Abbreviations

AAV: Adeno-associated virus
BMAL1: Brain and muscle ARNT-like protein 1
CLOCK: Circadian locomotor output cycles kaput
CRY: Cryptochrome
ERG: Electroretinogram
IPPTT: Intraperitoneal pyruvate tolerance test
OMR: Optomotor response behaviour
PER: Period
SCN: Suprachiasmatic nucleus
TH: Tyrosine hydroxylase
ZT: Zeitgeber time
