# Supplementary material for: Bmal1 overexpression in suprachiasmatic nucleus protects from retinal neurovascular deficits in a mouse model of diabetes
Source: Diabetologia. 2026 Apr 24;69(7):2088–100. doi: 10.1007/s00125-026-06734-1 (PMC13236736; doi:10.1007/s00125-026-06734-1)
Supplement: Supplementary file 1 — ESM (PDF 1244 KB) [file 125_2026_6734_MOESM1_ESM.pdf]

Electronic Supplementary Material

ESM Methods

2.1 Animals

All animals were maintained under normal physiological conditions (12 h light/ 12 h dark cycle, light intensity of about 325 lux, approximately 1m above the floor), with access to food (Teklad Irradiated Global 18% Protein Rodent Diet #2918, with energy density 3.1 kcal/g; energy from macronutrients- 24% from protein, 18% from fat, and 58% from carbohydrates) and water *ad libitum*. The zeitgeber time (ZT) indicates when lights turn on (ZT0=07:00 h) and off (ZT12=19:00 h) in animal facilities. The mice were randomly assigned to undergo stereotaxic surgery and receive viral delivery.

2.2 AAV-*Bmal1* delivery to the suprachiasmatic nucleus (SCN) and validation of *Bmal1* overexpression

AAV-Bmal1 sourcing

AAV-*Bmal1* was obtained from the Ocular Gene Therapy Core at the University of Florida, Gainesville, FL. The *Bmal1* gene was cloned under the smCBA promoter for ubiquitous expression, with a GFP tag, and subsequently packaged into AAV-8 for delivery and expression as smCBA-m*Bmal1*-P2A-GFP. The AAV8-smCBA-GFP was used as a control virus.

Stereotaxic delivery

Prior to all surgeries, mice were deeply anesthetized in an induction chamber using an isoflurane system (MGX Research Machine; Vetamac). Mice were then placed in a nose cone connected to the isoflurane system in an ultraprecise stereotaxic apparatus for rodents (900 series Ultraprecise

Kopf Instruments). DV was measured for lambda and bregma; nose cone was adjusted to within +/-0.05mm DV to ensure flatness of the skull.

For SCN overexpression, 8-weeks-old mice were injected with 0.4 nl/side with AAV-*Bmal1* or transfected with a control virus. Coordinates based on measurements from bregma were AP: -0.96 mm, ML: +/- 0.5 mm, and DV: -9.00 mm.

Quantification of Bmal1 overexpression

Bmal1 overexpression was quantified in *db/m* + *Bmal* and *db/db* + *Bmal* groups at study termination to validate optimum gene overexpression. A 1.0 mm diameter neural punch (Fine Science Tools) was used to acquire micropunches of the entire SCN. These micropunches occurred at rostrocaudal levels: -0.82 mm Bregma, -0.70 mm Bregma, -0.58 mm Bregma,-0.46 mm Bregma, and -0.34 mm Bregma, and non-SCN regions at 2.10 mm Bregma, 1.98 mm Bregma, 1.94 mm Bregma, and 1.78 mm Bregma (**ESM Fig. 1b**) were processed for mRNA expression of *Bmal1* as described below using qRT-PCR.

Total RNA was isolated using TRIzol reagent (Thermo Fisher Scientific) according to the manufacturer's protocol. RNA was reverse transcribed using cDNA Synthesis Kit (SuperScript VILO; Thermo Fisher Scientific). Gene-specific primers were used with a master mix (TaqMan Fast Universal; Thermo Fisher Scientific), and corresponding mRNA levels were determined by quantitative PCR (QuantStudio Real-Time PCR Systems; Thermo Fisher Scientific). All mRNA genes were normalized to *β-actin* and *TBP*, and data were analyzed using ΔΔCt method. The primers used were *β-actin* (Mm00607939_s1), *TBP* (Mm00446973_m1), and *Bmal1* (Mm00500226_m1).

2.3 Echo MRI

Live unanaesthetised mice were guided into a plastic tube designed to restrict their movement but not constrain them. The animals were then subjected to an imaging period (ZT3-ZT4) of 1-4 min in the gantry using a low-energy (0.05T) electromagnetic field in an Echo-MRI Body Composition Analyzer (E26-290-RMT, EchoMRI LLC, Texas, USA). Based on emitted T1 and T2 relaxation curves, lean mass, fat mass, free water, and total body weight are calculated using standard algorithms.

2.4 Wheel running activity

After 2 months of *Bmal1* overexpression, the mice were housed individually in cages (Phenome Technologies, Chicago, IL, USA) with running wheels in sound-attenuated and ventilated isolation cabinets with free access to water and food equipped with Actimetrics automated systems for animal behavioral testing (Actimetrics, Chicago, IL, USA) for 10 weeks, under the following segments (i) *Acclimatization (1-4 weeks;12 h light (300 lux):12 h dark)*: The animals were acclimatized in the circadian rhythm cabinets for four weeks (ii) *Wheel running activity (5-6 weeks;12 h light (300 lux): 12 h dark):* The average wheel-running activity was recorded. The total wheel-running activity was analyzed with Clocklab (Actimetrics, Chicago, IL, USA) as an average over a one-week window. (iii) *Free Running Period (7-10 weeks; constant dark)*: The lighting conditions were switched to constant dark for the remainder of the study. The free-running period (τ) under constant dark conditions was calculated as the average over a two-week window, following at least 10 days of exposure under constant dark conditions.

2.5 Neuronal function using electroretinogram (ERG)

All animals were dark-adapted overnight prior to ERG recordings. The mice were anesthetized by administering an i.p. injection of ketamine (100 mg/kg) and xylazine (5 mg/kg). The pupils were

dilated using a topical application of 1% tropicamide and 2.5% phenylephrine (Alcon Laboratories). The eyes were kept moist using a 2.5% hypromellose ophthalmic demulcent solution/Gonak (Akorn). For the ERG recordings, the ground needle electrode was placed on the base of the tail, and the reference electrode was subdermally placed between the eyes. The gold loop electrodes (LKC Technologies, Inc., Gaithersburg, MD, USA) placed over the cornea were used to record the ERG response. The stimulus flash intensities of 0.025, 0.25, and 2.5 cd x s/m^2 were presented in a UTAS ganzfeld illuminator (LKC Technologies) under scotopic conditions. The values for a-wave and b-wave amplitudes and their implicit times were obtained from an inbuilt analysis tool by LKC Technologies.

2.6 Optomotor response behavior (OMR)

Spatial vision was assessed by measuring the spatial-frequency threshold for optometer response using an OptoMotry device (CerebralMechanics, Inc.). Tracking head movements in response to rotating sine-wave gratings (100% contrast) was recorded in free-moving mice. Spatial frequency was systematically increased using a staircase method until the animal failed to respond, and the highest spatial frequency it could track was taken as the threshold. The threshold obtained for each eye was reported.

2.7 Immunohistochemistry for tyrosine hydroxylase (TH)

The liver samples were fixed in 4% PFA in PBS for 24 h, then embedded in paraffin. The sections were placed on charged slides and dried overnight at 56°C. Slides were subsequently deparaffinized in xylene and hydrated through descending grades of ethyl alcohol to distilled water, and then placed in Tris-Buffered Saline (pH 7.4) (Scytek Labs, Logan, UT) for 5 min to adjust the pH. The sections were then subjected to enzyme-induced epitope retrieval in 0.03% Pronase E/TBS (Millipore Sigma/Scytek) in an incubator at 37°C for 10 min, followed by several

rinses in distilled water. Before proceeding with blocking for non-specific proteins with rodent Block M (Biocare, Concord, CA) for 20 min, the sections were pre-treated with 3% hydrogen peroxide/methanol for 30 min at 25°C, rinsed with distilled water, and washed with TBST for 5 min, followed by micro-polymer staining performed at room temperature on the Biocare intelliPATH automated stainer. Lastly, the sections were incubated with primary antibody (rabbit Tyrosine Hydroxylase (Catalog #: AB152, Millipore Sigma, Temecula, CA) at 1:150 in Normal Antibody Diluent (Scytek) for 1 h, followed by rodent HRP Polymer (Biocare) incubated for 30 min. Reaction development utilized Romulin AEC (Biocare) for 5 min, counterstained in CATHE Hematoxylin diluted 1:10 for 1 min, followed by air drying, dipping in xylene, and coverslipping with permanent mounting media. The slides were imaged at 20X under a fluorescent microscope (Zeiss AXIO Observer A1 Inverted Fluorescence Microscope, Carl Zeiss MicroImaging GmbH).

2.8 Vascular deficits

The whole eyes were fixed in 4% paraformaldehyde. The day before trypsin digestion, the retinas were isolated and placed in 50 ml of water for overnight unfixing. The individual retina was incubated in 3% trypsin at 37°C for 2 h the next day. The trypsin-digested retina was placed in a petri dish, and the internal limiting membrane was gently separated from the peripheral retina with fine forceps. Then, using Vannas's scissors, the internal limiting membrane was isolated from the optic nerve. Subsequently, the neural retina was removed, and the isolated retinal vasculature was stained with periodic acid and Schiff's base to assess acellular capillary numbers.

2.9 Norepinephrine levels using ELISA

Whole blood was collected in an EDTA-coated collection tube during the animal sacrifice. After

30 min, the blood samples were centrifuged at 2000 x g for 20 min. The clear supernatant or plasma was separated and stored at -80˚C. Plasma norepinephrine levels were quantified using a commercially available norepinephrine ELISA kit [cat no. 3836; Novus Biologicals LLC, CO, USA] as per the manufacturer's guidelines.

2.10 Intraperitoneal glucose tolerance test (IPGTT)

The animals were fasted for 4 h, and basal blood glucose levels were monitored with a commercially available glucometer (AlphaTrek2) to determine the basal (zero-time) blood glucose level. After which, a glucose solution (1g/kg body weight) was administered *via* the i.p. route. The blood glucose was then measured again at 10, 20, 30-, 60-, 90-, and 120 min post-glucose administration.

2.11 Intraperitoneal insulin tolerance test (IPITT)

The animals were fasted for 2 h for ITT, and basal blood glucose levels were quantified as described above. An i.p. injection of 0.5 IU/kg insulin Humulin R U-100 was administered to the animals, followed by measurement of glucose levels as described in IPGTT.

2.12 Intraperitoneal pyruvate tolerance test (IPPTT)

The animals were fasted for 16 h before basal blood glucose measurements, and sodium pyruvate (P5280, Millipore Sigma, US), prepared in sterile PBS at 1g/kg body weight, was administered intraperitoneally. The blood glucose levels were subsequently quantified using a glucometer at 15, 30, 60, 90, and 120 min, as described earlier.

Supplementary Figures

ESM Fig. 1

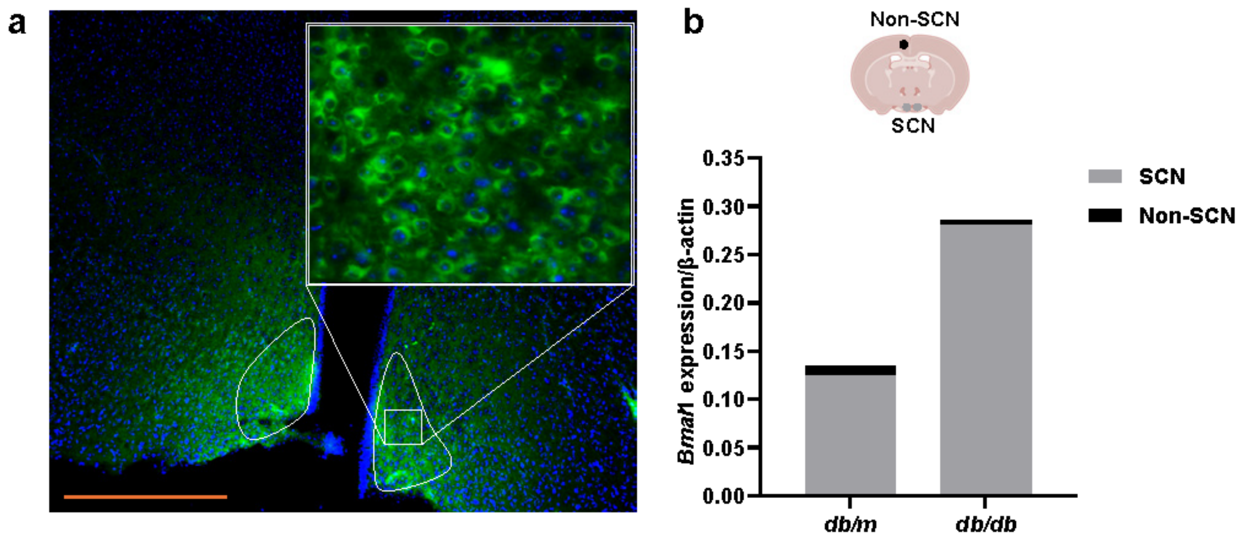

Fig. 1: **Expression of AAV-*Bmal1* in the SCN of hypothalamus.** Mice were injected with AAV-*Bmal1* stereotaxically, four weeks post-injections, the SCN region of the hypothalamus was stained to locate the overexpression of *Bmal1*. **(a)** Representative images showing *Bmal1* expression in the SCN. Magnification: 20X and Scale Bar- 100μM. **(b)** *Bmal1* expression was analyzed in the SCN and non-SCN regions of *db/m* + *Bmal* and *db/db* + *Bmal* mice at study termination. The *Bmal1* expression was significantly higher in *db/db* +*Bmal* mice (p=0.05); n= 3. The data is presented as Mean ± SEM and analyzed using the Mann-Whitney test.

ESM Fig. 2

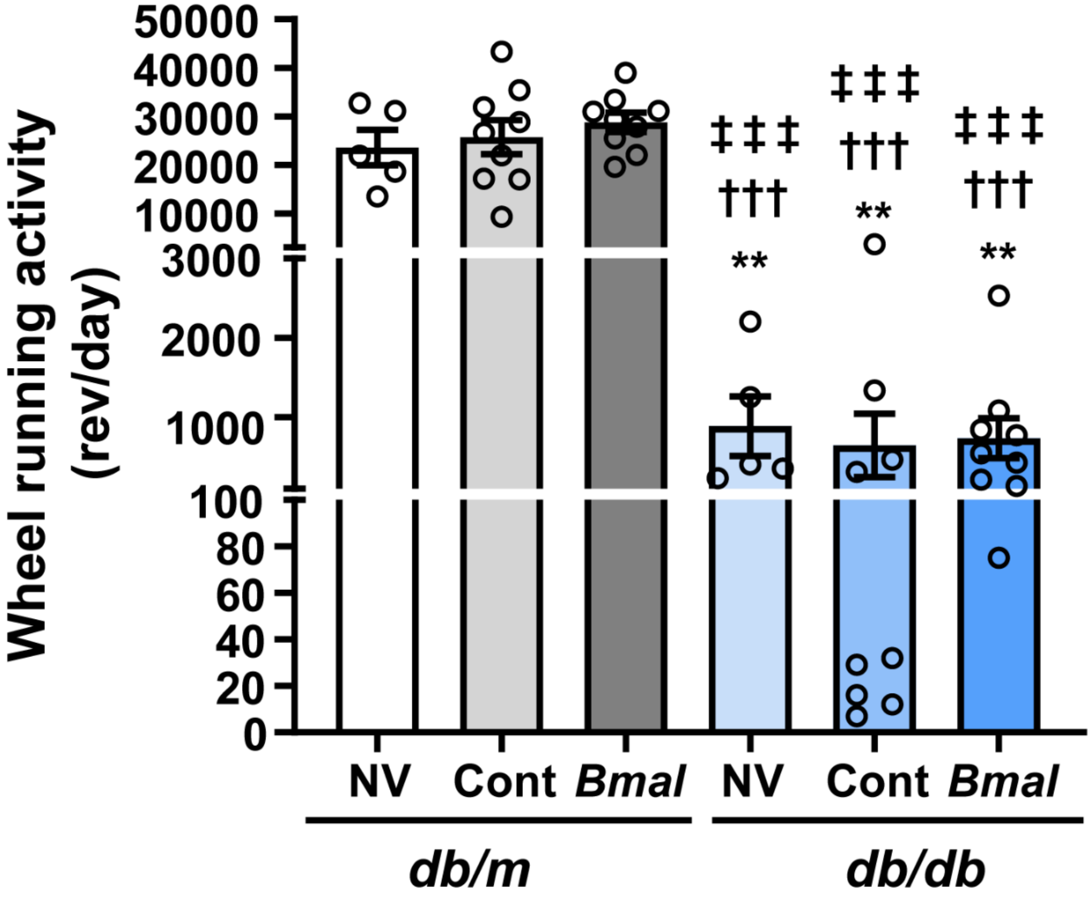

Fig. 2: Effect of *Bmal1* overexpression on wheel running activity. Bar chart showing total wheel-running activity across different conditions. The number of animals in each group is as follows: *db/m* + NV: *n*=5; *db/m* + Cont: *n*=9; *db/m* + *Bmal:* *n*=9; *db/db* + NV: n=5; *db/db* + Cont: n=9; *db/db* + *Bmal:* *n*=9. The data is presented as Mean ± SEM and analyzed using Brown-Forsythe and Welch ANOVA test followed by unpaired t-test with Welch's correction; * vs *db/m* + NV, ** = $p < 0.01$; † vs *db/m* + Cont, ††† = $p < 0.001$; ‡ vs *db/m* + *Bmal*, ‡‡‡ = $p < 0.001$.

ESM Fig. 3

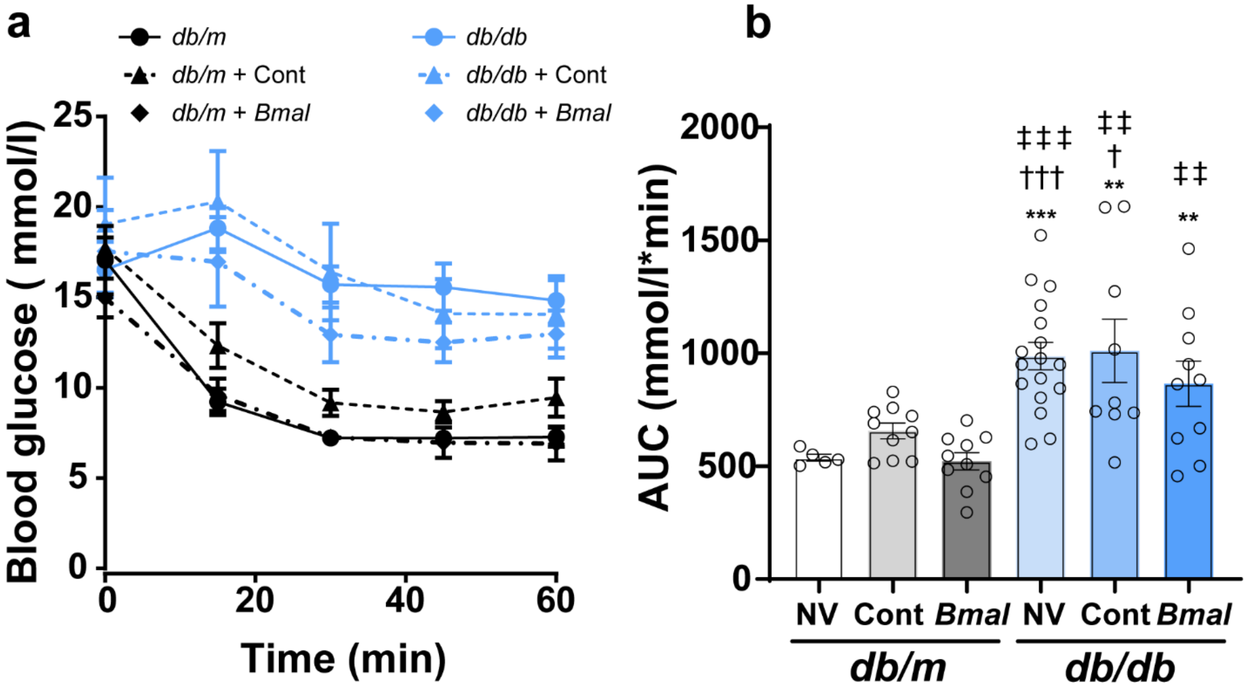

Fig. 3: Effect of *Bmal1* overexpression on insulin sensitivity in *db/db* mice. The mice were fasted for 2 h, followed by an i.p. injection of insulin at 0.5 IU/kg/body weight. Blood glucose was monitored using a commercially available glucometer. **(a)** IPITT and its **(b)** AUC in the respective groups. *n* in each group is as follows: *db/m* + NV: *n*=5; *db/m* + Cont: *n*=10; *db/m* + *Bmal:* *n*=10; *db/db* + NV: *n*=17; *db/db* + Cont: *n*=9; *db/db* + *Bmal:* *n*=10. The data is presented as Mean ± SEM and analyzed using Brown-Forsythe and Welch ANOVA test followed by unpaired t-test with Welch's correction; * vs *db/m* + NV, ** = p<0.01, *** = p<0.001; † vs *db/m* + Cont, † = p< 0.05, ††† = p< 0.001; ‡ vs *db/m* + *Bmal*, ‡‡ = p<0.01; ‡‡‡ = p< 0.001.
